# The Dark Side of Melanin Secretion in Cutaneous Melanoma Aggressiveness

**DOI:** 10.3389/fonc.2022.887366

**Published:** 2022-05-10

**Authors:** Luís C. Cabaço, Ana Tomás, Marta Pojo, Duarte C. Barral

**Affiliations:** ^1^ Chronic Diseases Research Center (CEDOC), NOVA Medical School, NMS, Universidade NOVA de Lisboa, Lisbon, Portugal; ^2^ Unidade de Investigação em Patobiologia Molecular (UIPM), Instituto Português de Oncologia de Lisboa Francisco Gentil E.P.E., Lisbon, Portugal

**Keywords:** cutaneous melanoma, melanin, ultraviolet radiation, melanogenesis, melanin secretion, microenvironment, immunomodulation, aggressiveness

## Abstract

Skin cancers are among the most common cancers worldwide and are increasingly prevalent. Cutaneous melanoma (CM) is characterized by the malignant transformation of melanocytes in the epidermis. Although CM shows lower incidence than other skin cancers, it is the most aggressive and responsible for the vast majority of skin cancer-related deaths. Indeed, 75% of patients present with invasive or metastatic tumors, even after surgical excision. In CM, the photoprotective pigment melanin, which is produced by melanocytes, plays a central role in the pathology of the disease. Melanin absorbs ultraviolet radiation and scavenges reactive oxygen/nitrogen species (ROS/RNS) resulting from the radiation exposure. However, the scavenged ROS/RNS modify melanin and lead to the induction of signature DNA damage in CM cells, namely cyclobutane pyrimidine dimers, which are known to promote CM immortalization and carcinogenesis. Despite triggering the malignant transformation of melanocytes and promoting initial tumor growth, the presence of melanin inside CM cells is described to negatively regulate their invasiveness by increasing cell stiffness and reducing elasticity. Emerging evidence also indicates that melanin secreted from CM cells is required for the immunomodulation of tumor microenvironment. Indeed, melanin transforms dermal fibroblasts in cancer-associated fibroblasts, suppresses the immune system and promotes tumor angiogenesis, thus sustaining CM progression and metastasis. Here, we review the current knowledge on the role of melanin secretion in CM aggressiveness and the molecular machinery involved, as well as the impact in tumor microenvironment and immune responses. A better understanding of this role and the molecular players involved could enable the modulation of melanin secretion to become a therapeutic strategy to impair CM invasion and metastasis and, hence, reduce the burden of CM-associated deaths.

## Introduction

CM is an increasingly concerning public health issue, mainly due to its rising incidence, fast metastasis and high mortality rates (1). CM arises from the malignant transformation of melanocytes found in the skin epidermis, which produce the photoprotective pigment melanin and are also involved in ROS/RNS scavenging, thermoregulation and immunomodulation (2–4). Even though it only represents 5% of all types of skin neoplasms, CM is considered the most lethal, being responsible for 60-75% of skin cancer-related deaths (5–7). From 1990 to 2019, incident cases of CM have increased by 170% worldwide, faster than most neoplasms, mainly due to a rapidly aging population, changes in lifestyle and better screening (8, 9). Furthermore, CM has a higher potential to form metastasis in surrounding tissues than other cancers (10), which vastly contributes to its lethality and extremely poor prognosis. In 2020 alone, there were almost 60,000 CM-related deaths worldwide and more than 300,000 new cases (11). Currently, CM is classified based on tumor thickness, presence or absence of ulceration, lymph node involvement, and presence or absence of distant metastases (12). Whilst stage I and II CM do not form metastases, stage III CM presents with lymph node involvement, and stage IV CM with distant metastases (12). Patient prognosis varies greatly depending on the stage – stage I CM is characterized by a 5-year survival rate of 99.4%, which drops to only 23% in patients with stage IV CM (13). Thus, early detection of CM is crucial to improve prognosis and patient survival.

Over recent years, advancements in the molecular biology of CM have highlighted the relevance of the genomic profile of CM for accurate prognosis and disease management (2). The most frequently altered signaling pathway in CM is the Mitogen-activated protein kinase/Extracellular signal-regulated kinase (MAPK/ERK) pathway, with ~52% of patients displaying mutations in the *BRAF* proto-oncogene - encoding a serine/threonine kinase - most commonly V600E (14). Curiously, even though *BRAF* mutations drive CM growth and proliferation, they have also been identified in a large percentage of benign nevi, and are insufficient *per se* to induce oncogenesis (15, 16). Patients can also harbor other alterations in this pathway, such as mutations in the proto-oncogene *NRAS*, encoding a small GTPase, and *Neurofibromin 1* (*NF1*) (~28% and ~14%, respectively). This ultimately leads to the constitutive activation of the MAPK/ERK pathway, promoting tumor cell survival and proliferation. Furthermore, activating alterations in the Phosphatidylinositol-4,5-bisphosphate 3-kinase/protein kinase B (PI3K/AKT) pathway are also common (2, 14).

Additionally, targeted therapies and immunotherapies have greatly improved CM treatment, significantly increasing the overall survival of CM patients (2, 17–19). Despite surgical excision being sufficient in cases where CM is detected at an early stage, in advanced stages (III and IV), the disease becomes disseminated and cannot be eliminated by excision alone (2, 20). In the cases where *BRAF* mutations are present, selective BRAF and Mitogen-activated protein kinase (MEK) inhibitors improve patient clinical outcomes (17, 18, 21). However, acquired resistance is common and only half of the patients seem to benefit from targeted therapies (18, 22). Regarding immunotherapies, immune-checkpoint inhibitors anti-Programmed cell death protein 1 (anti-PD-1 - nivolumab, pembrolizumab) and anti-Cytotoxic T-lymphocyte-associated protein 4 (anti-CTLA4 - ipilimumab) have been shown to also improve patient overall survival, leading to long lasting responses (17, 20). In fact, due to its high mutational burden, CM is considered one of the most immunogenic types of tumors, able to modulate the immune microenvironment to its advantage and favoring immune evasion and suppression (23). Hence, immunotherapies have great potential to overcome these tumor advantages by releasing effector T cell suppression, allowing T lymphocytes to eliminate CM cells (23). Recently, the 5-year overall survival of patients receiving a combination of nivolumab and ipilimumab was documented to be 52% (24). Immunotherapy is therefore considered the first-line option for treatment of stage III and IV CM (20). Still, 40-65% of patients show primary resistance to these therapies, and 20-30% of responding patients end up developing secondary resistance (17, 25–27). Moreover, the costs associated with immunotherapies are high, which can limit access to these treatments, leading to under treatment (28). Thus, there is an urgent need for new and improved therapeutic approaches to decrease the overall global CM burden.

## The Role of Melanin in Cutaneous Melanoma Progression

**Figure 1:**
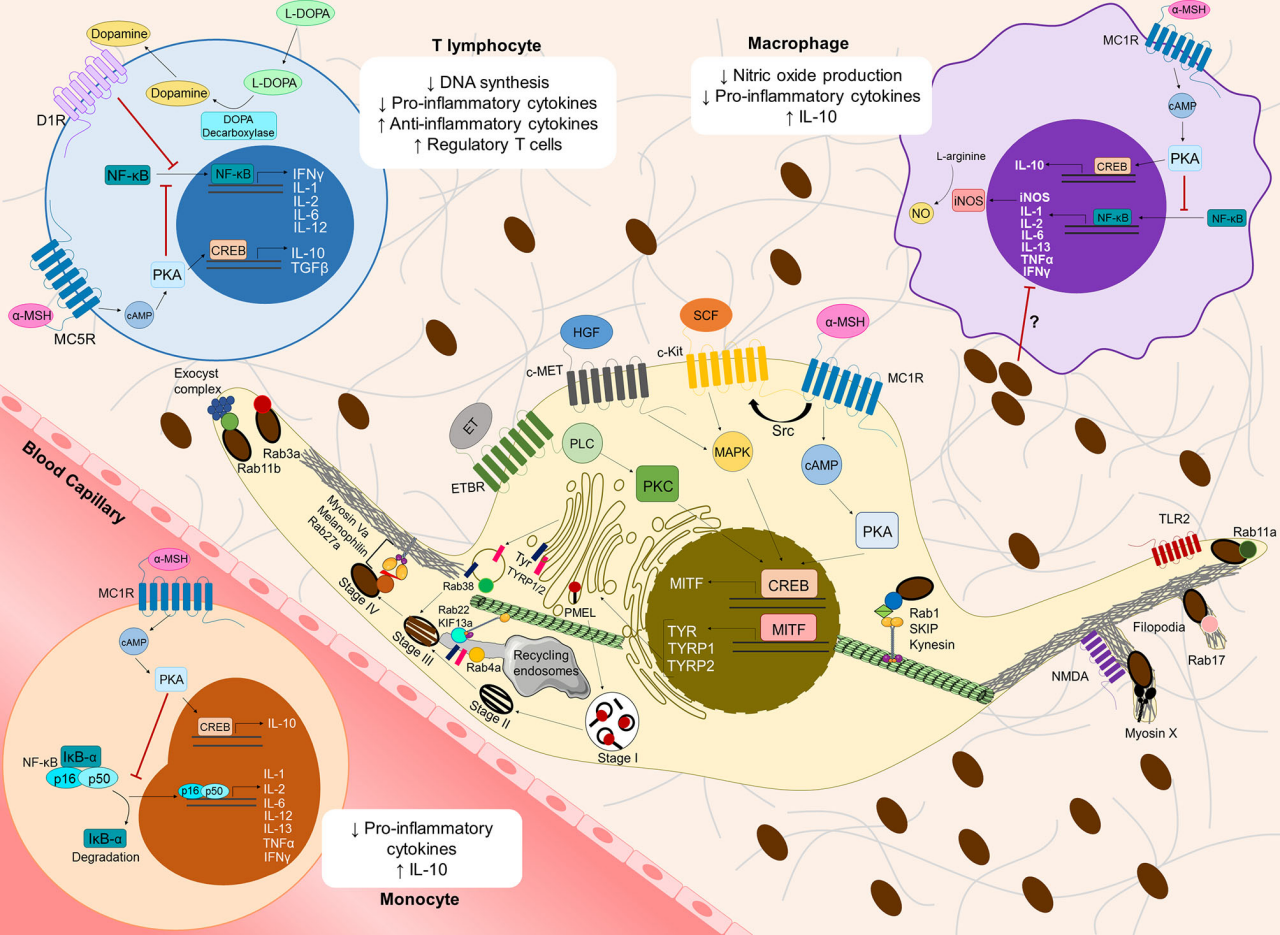
Overview of cutaneous melanoma-related molecular mechanisms involved in melanin synthesis, transport and secretion, and their contribution to an immunosuppressive microenvironment. Several receptors at the plasma membrane [Melanocortin 1 receptor (MC1R), c-Kit receptor tyrosine kinase (c-Kit), c-Met receptor tyrosine kinase (c-Met) and Endothelin receptor type B (ETBR)] recognize distinct keratinocyte/fibroblast-derived soluble factors [α-Melanocyte stimulating hormone (α-MSH), Stem-cell factor (SCF), Hepatocyte growth factor (HGF) and Endothelin (ET)], inducing several signaling pathways, which in turn activate the cAMP response element-binding protein (CREB) and subsequently the Microphthalmia-associated transcription factor (MITF). MC1R can transactivate c-Kit receptor by recruiting the tyrosine kinase Src. MITF stimulates the synthesis of the melanogenic enzymes Tyrosinase (Tyr) and Tyrosinase-related protein 1/2 (TYRP1/2), which are transported from the Golgi to melanosomes to initiate melanogenesis. Melanin production requires melanosome maturation, a process that can be divided in four different stages (I to IV). Stage I melanosomes are early endosome-derived organelles containing intraluminal vesicles, which serve as platforms for the deposition of the glycoprotein PMEL. PMEL is later cleaved to form the internal fibrillar striations of stage II melanosomes, giving them an elliptical shape. Then, Tyr and TYRP1/2 are delivered to stage II melanosomes in Golgi-derived Rab38-positive vesicles. Moreover, Tyr and TYRP1/2 can also be delivered from recycling endosomes in a Rab4a-dependent manner and/or through tubular extensions dependent on the complex Rab22/KIF13a. In stage III melanosomes, melanin starts to deposit onto PMEL fibrils and melanosomes reach full maturation in stage IV. Melanosomes interact with the cytoskeleton *via* two tripartite complexes: Rab1/SKIP/Kinesin on microtubules and Rab27a/Melanophilin/MyosinVa on the cortical actin network at the melanocyte periphery. For melanosome secretion, Rab11b interacts with the exocyst tethering complex, while Rab11a is involved in melanin secretion mediated by Toll-like receptor 2 (TLR2). Moreover, Rab3a regulates melanin exocytosis induced by keratinocyte-conditioned medium. Myosin X, Rab17 and N-methyl-D-aspartate (NMDA) receptor are involved in melanin release through filopodia. The molecular regulators described above participate in cutaneous melanoma (CM) proliferation, migration, invasion and/or metastasis (see **Supplementary Table 1**). Melanogenesis intermediates also suppress the immune microenvironment, namely by modulating cytokine expression in monocytes, macrophages and T lymphocytes. The binding of α-MSH to MC1R at the surface of circulating monocytes or local macrophages, as well as its recognition by MC5R at the surface of T lymphocytes activates Protein kinase A (PKA) though cAMP, which in turn inhibits Nuclear factor kappa B (NF-κB) translocation to the nucleus. Consequently, the translation of several pro-inflammatory cytokines is inhibited. Furthermore, α-MSH induces the production of anti-inflammatory cytokines (IL-10, TGFβ), possibly through CREB activation, thereby increasing the infiltration of regulatory T cells in the CM microenvironment. In macrophages, inhibition of NF-κB by α-MSH also decreases the levels of nitric oxide synthase (iNOS), which in turn inhibits nitric oxide production. L-DOPA has been shown to decrease DNA synthesis and pro-inflammatory cytokine production in T lymphocytes, but molecular mechanisms behind it remain mostly unknown. Interestingly, T lymphocytes can express DOPA decarboxylase, which converts L-DOPA into dopamine. Through autocrine signaling, dopamine can bind to dopamine receptors at the surface of T lymphocytes (*e.g.* D1R) and suppress these cells. Melanin itself might also suppress pro-inflammatory cytokine production in monocytes/macrophages, but the molecular mechanisms underlying this process remain elusive.

Melanocytes are specialized cells responsible for the synthesis, packaging and transfer of melanin to neighboring keratinocytes in the skin epidermis (29–31). The role of melanin in CM remains controversial, despite it being extensively studied and considered unquestionably involved in the progression of this type of cancer. This is probably due to melanin behaving as a “double-edged sword”. Indeed, melanin has photoprotective functions, as it absorbs ultraviolet radiation (UVr) and scavenges ROS/RNS resulting from this exposure (32). However, the scavenged ROS/RNS were found to modify melanin and contribute to mutations triggering CM cell immortalization and carcinogenesis (33). Moreover, changes in the molecular machinery responsible for regulating melanin synthesis in melanocytes have been linked to the phenotypic switch from proliferative to invasive CM states (**Figure 1** and **Supplementary Table 1**) (29, 30). In contrast, it has been described that the presence of melanin inside CM cells decreases their invasiveness by increasing cell stiffness and reducing elasticity (34, 35). Interestingly, several regulators of melanin secretion have also been found altered in CM (**Figure 1** and **Supplementary Table 1**), and in advanced CM stages melanin can be found in the dermis, where it contributes to microenvironment modulation and CM progression (36–40).

## Melanin: A “Double-Edged Sword” in Cutaneous Melanoma Initiation

Melanin is the pigment responsible for skin photoprotection against solar UVr-induced damage (32, 41–43). Supporting this protective role of melanin, skin phototypes are darker in equatorial and tropical regions of our planet, where UVr is more intense (32). Despite its protective role, melanin can become harmful. Indeed, ROS/RNS generated by UVr induce the formation of cyclobutane pyrimidine dimers (CPDs), even hours after UVr exposure (33, 44). In part, CPDs are directly induced by UVB exposure but a major part of DNA lesions is induced by modified melanin (33, 44). Importantly, CPDs are known as signature DNA lesions in CM cells and require UVr and oxidized melanin to form (33, 44). In agreement with this notion, epidemiological studies have shown that dark skin individuals display a much higher frequency of CM and a lower frequency of other non-CM skin cancers, when compared to albinos, in which CM is very rare and carcinomas occur frequently (45, 46). Therefore, these studies suggest that melanin is required for the development of CM. Another study compared light and dark skin individuals living in South Africa and found that CM incidence in the latter represents only 10% of the total incidence observed in the caucasian population (47). In addition, another study found that lightly or intermediately-pigmented skin explants irradiated with high UVr doses have ≥ 80% of melanocytes containing CPDs, while only 15% of melanocytes in darkly-pigmented skin explants show CPDs upon the same irradiation conditions (48–50). Altogether, these studies raise the importance of melanin in CM initiation and show that melanin in lightly pigmented skins presents distinct biochemical and physical properties, being more prone to contribute to CM pathology than melanin in darkly pigmented skins.

Melanocytes produce two types of melanin in lysosome-related organelles called melanosomes, namely black/brown eumelanin and yellow/red pheomelanin (32, 51). Importantly, the type of melanin is also a key determinant of skin pigmentation, in addition to its amount (32). Although both types of melanin may be produced by a single melanocyte, dark skin individuals produce more eumelanin, while light skin individuals have increased levels of pheomelanin (52). Both eumelanin and pheomelanin require the same precursors - L-tyrosine or L-DOPA - but differ on the presence and activity of specific melanogenic enzymes and intermediate substrates (32, 53). Therefore, after the oxidation of tyrosine or DOPA to DOPAquinone by the key melanogenic enzyme Tyrosinase (Tyr), two distinct outcomes are possible (32, 53). In the presence of the amino acid cysteine, pheomelanogenesis is favored, while when cysteine is consumed in melanocytes, eumelanogenesis takes place (51, 53, 54). Even though the mechanism is not fully understood, the concentration of cysteine is strictly regulated by the Major Facilitator Superfamily Domain Containing 12 (MFSD12) importer and the cystinosin exporter channels located at the membrane of melanosomes (53, 55, 56). Eumelanosomes and pheomelanosomes have different composition and structure (57, 58). The most striking difference in the context of CM is the oxidative potential observed in the presence of pheomelanin, contrasting with the reduction potential of eumelanosomes (57). Indeed, pheomelanin synthesis produces higher levels of ROS/RNS that can be harmful for melanocytes and may lead to CM initiation, even without UVr exposure (59, 60). In general, pheomelanin is considered phototoxic, while eumelanin is photoprotective (57, 61). Importantly, both Tyrosinase-related protein 1 (TYRP1) and 2 (TYRP2) enzymes control eumelanogenesis rate, by fine tuning Tyr activity, even though they are not required for pheomelanogenesis. Additionally, Tyr activity is lower in pheomelanogenesis. Interestingly, Tyr has higher activity in the slightly acidic lumen of melanosomes, whose pH is strictly controlled by different ion channels located at the melanosome membrane, including vacuolar proton ATPases, copper importers, sodium/proton exchangers, potassium-dependent calcium/sodium exchangers, and NAADP-dependent calcium exporters (51, 53, 55, 62). The ion channel crosstalk at the melanosome membrane, the different activity and presence/absence of melanogenic enzymes, as well as different melanosome properties and organization were observed in eumelanosomes and pheomelanosomes. These and other molecular mechanisms should be further studied to better understand what determines if melanin has harmful or protective properties and how this quality control is done by melanocytes in order to avoid CM initiation and progression.

Besides the duality previously mentioned concerning photoprotection and UVr-induced photosensitization, melanin can contribute to the pathology of CM in other ways. Indeed, melanin has been shown as an immunomodulator biopolymer and it is known that CM involves a high burden of immunosuppression associated with the tumor microenvironment (33, 63). Langerhans cells, macrophages, monocytes, T lymphocytes and mast cells are important players in skin homeostasis and pigmentation (64, 65). Specifically, macrophages are implicated in skin integrity, by protecting against external pathogens, and also in wound repair and ion balance (65). Mast cells also regulate the skin barrier, mainly by enhancing fibroblast growth and vascular development through growth factor secretion (66). Furthermore, immune cells in the skin microenvironment release soluble factors that can impact skin pigmentation, especially during inflammation triggered by UVr, pathogens or chemicals (64, 67). For instance, Langerhans cells, monocytes and macrophages are able to produce Interleukin (IL)-18, a cytokine that increases Tyr expression, upregulating melanin biosynthesis (68). Granulocyte macrophage-colony stimulating factor (GM-CSF) and IL-33 secreted by macrophages or T lymphocytes, and mast cells, respectively, also promote melanogenesis (65, 69). Conversely, T lymphocytes, macrophages, and monocytes are able to produce Interferon (IFN)-γ, IL-17, and Tumor necrosis factor (TNF), which have been shown to prevent melanosome maturation and decrease Tyr levels (70, 71). IL-4 secreted by T lymphocytes can also decrease Tyr, TYRP1, and TYRP2 expression *via* the JAK2-STAT6 signaling axis (72).

On the other hand, melanin is known to suppress the production of pro-inflammatory cytokines by T lymphocytes, monocytes, macrophages, fibroblasts and endothelial cells (73–76). Moreover, UVr-induced melanin chemiexcitation enhances the production of α,β-unsaturated melanin-carbonyl and RNS, which have been shown to inhibit the immune response of T lymphocytes in CM microenvironment (33, 77, 78). In fact, CM is an immunogenic type of tumor in which the immune system in the tumor microenvironment is manipulated and has a crucial role in CM progression (79, 80). Moreover, melanin may have an important role in this immunomodulation. Interestingly, in mice with abnormal expansion of melanocytes in the dermis, *i.e.* dermal melanocytosis, melanin seems to be captured and transported to regional lymph nodes by Langerhans cells (81, 82). Although the role of melanin in CM initiation is well described, its role in advanced CM stages is poorly understood. However, it needs to be the subject of future studies because the immunomodulatory properties of melanin raise a crucial role for melanin in CM progression.

## Melanogenesis Machinery Contributes to Cutaneous Melanoma Progression

Melanin plays an essential role in CM pathology through the malignant transformation of melanocytes (32, 33, 41–44). Skin pigmentation is maintained by the crosstalk between the main cell types of the skin, namely melanocytes, keratinocytes, fibroblasts, and immune cells such as macrophages and mast cells. Factors released by keratinocytes and fibroblasts upon UVr exposure stimulate melanin synthesis by melanocytes, in a process known as adaptive pigmentation or “tanning”. These factors include α-Melanocyte stimulating hormone (α-MSH), which is released by keratinocytes and binds to Melanocortin-1 receptor (MC1R) on the surface of melanocytes, activating a signaling pathway dependent on the secondary messenger Cyclic adenosine monophosphate (cAMP). This leads to an increase in the levels of Melanocyte inducing transcription factor (MITF), the master regulator of the expression of melanogenic enzymes like Tyr, as well as melanocyte dendricity, proliferation and differentiation (83, 84). Moreover, keratinocyte- and fibroblast-derived soluble factors can bind to specific receptors on the surface of melanocytes and lead to the activation of MITF by inducing downstream signaling pathways. Interestingly, some of these soluble factors or their receptors were reported to have a role in CM initiation and progression (**Figure 1** and **Supplementary Table 1**). For instance, *MC1R* is a highly polymorphic gene that can shift the ratio of eumelanin/pheomelanin depending on single-nucleotide polymorphisms (SNPs) present in melanocytes (85). Some of these SNPs, especially those favoring pheomelanin production, are associated with an increased risk of CM (86, 87). MC1R is associated with CM proliferation and survival through the MAPK/ERK-signaling pathway, as well as with increased migratory capacity, by controlling the expression of the transmembrane protein Sidecan-2 (88–90). MC1R was also reported to transactivate the tyrosine kinase c-Kit receptor, which is activated by the Stem-cell factor (SCF) derived from skin keratinocytes and fibroblasts (89). Upon activation, oncogenic c-Kit receptor mutants stimulate CM proliferation and survival through MAPK/ERK and PI3K/AKT signaling pathways (91–93). Another keratinocyte-derived soluble factor involved in CM progression is Endothelin (ET), which induces CM proliferation and survival; neoangiogenesis by upregulation of Vascular endothelial growth factor (VEGF); migration through increased expression of αvβ3 and α2β1 integrins and focal adhesion kinase overactivity; epithelial-mesenchymal transition (EMT) by switching the expression of E-cadherin to N-cadherin; and invasion by enhanced activity of Matrix metalloproteinases (MMPs) (94–98). Additionally, hepatocyte growth factor, which is released by both keratinocytes and fibroblasts upon UVr exposure, binds to its receptor (c-Met) at the plasma membrane of CM cells and increases proliferation and survival through the MAPK/ERK pathway, as well as migration and invasion through ROS-mediated signaling (99, 100). These are only a few examples of keratinocyte- and fibroblast-derived molecules that can be recognized by CM cells and disrupt the signaling pathways mentioned above, in order to favor their pathologic transition. Importantly, the crosstalk between the signaling pathways linked with these receptors and the ability to produce several of these soluble factors in advanced CM stages, can contribute to autocrine regulation of CM progression and entail CM plasticity and increased drug-resistance (101). In addition to intercellular communication through soluble factors, other interactions are crucial for CM progression. CM cells were shown to locally change the differentiation pattern of keratinocytes *in vitro* and *in vivo*, usually by aberrant expression of basal Keratin-14 in suprabasal epidermal layers, accompanied by loss of Keratin-10 (102, 103). Importantly, the expression of typical keratins from the bottom two layers of epidermis, namely basal and spinous layers, are associated with low overall survival in CM patients (104). Indeed, skin function and homeostasis are not only modulated by melanocyte and CM cell capacity to produce and transfer melanin. Upon stress conditions like UVr-exposure, melanocytes also enhance the secretion of several soluble factors such as cytokines, extracellular matrix components, classical neurotransmitters, neuropeptides and hormones (52, 105–110). Interestingly, after UVr-exposure (specially UVB), the neuroendocrine property of skin cells is crucial to locally mediate an homeostatic response, and release soluble factors into blood/lymph circulation. This humoral response also activates the hypothalamic-pituitary-adrenal (HPA) axis, composed by the global homeostatic regulatory elements hypothalamus, pituitary, adrenal glands, and immune system (52, 111, 112). This two-way communication between the brain and skin occurs mainly due to the ability of melanocytes and CM cells not only to produce and secrete several regulators of the HPA axis, but also to express their receptors on the cell surface and hence, exert an autocrine, paracrine and intracrine regulation of their functions. Moreover, these HPA axis regulators follow a production and expression hierarchy, as observed in the HPA axis, namely Corticotropin-releasing factor (CRF) → Pro-opiomelanocortin (POMC) → POMC-derived peptides such as Adrenocorticotropic hormone (ACTH), β-Endorphin, and α-, β- and γ-MSH (52, 107, 111, 113, 114). Noteworthy, POMC and POMC-derived peptides show increased expression from nevi to advanced CM cells, indicating their crucial role in CM progression. CM can subvert the stress sensory capacity and neuroendocrine functions to modulate melanogenic activity, suppress local and systemic immune responses, increase CM tumor survival in its microenvironment, and therefore, favor CM cell growth and invasiveness (112, 115–118).

Not surprisingly, as the master regulator of several melanocyte functions including melanogenesis, MITF is also a key player in CM initiation and progression (**Supplementary Table 1**). Generally, in early CM stages, there is a hyperactivation of MITF and CM cells display dysregulated melanin synthesis, accumulating the pigment intracellularly and leading to heavy pigmentation, as well as a high proliferative rate. MITF activation is then progressively reduced from early to advanced CM stages, being involved in the phenotypic switch from proliferation to invasion (29, 30, 105, 119–121). However, CM cells with high or low MITF levels coexist in CM tumors, which can originate from a reversible phenotypic switch that is responsible for intratumor heterogeneity, CM plasticity and increased therapeutic resistance (119, 122–125). This complexity of CM could explain the controversial results reported in the literature. For instance, MITF repression was shown to reduce proliferation, as well as migration and invasion by increasing the number of focal adhesions, while inducing EMT (126). Oncogenic BRAF, which favors CM proliferation, is found in approximately 52% of human CMs (14), and was recently shown to suppress MITF expression. Despite MITF being usually found to promote proliferation, when MITF expression is enhanced in BRAF-mutated CM cells, their proliferation is inhibited (127). MITF activation is also associated with increased melanogenesis. Indeed, many melanogenic substrates and enzymes, as well as some melanosomal markers have been associated with CM progression (**Supplementary Table 1**). The ratio between the melanogenic substrates L-DOPA/L-tyrosine is increased in the serum of patients with invasive and metastatic CM and higher in patients with evolutive disease, compared with stable patients (128). Curiously, both L-tyrosine and L-DOPA have a role as “hormone-like” signaling molecules in melanocytes, besides functioning as melanogenic substrates (129). They were shown to bind to plasma membrane and nuclear envelope proteins, stimulating the expression of POMC, POMC-derived peptides and MSH receptors, without changing insulin binding capacity (129–133). This illustrates the receptor-dependent regulatory capacity of melanogenic substrates on CM cell functions during disease progression. Moreover, these two melanogenic substrates can change melanocyte and CM cell metabolism in a receptor-independent manner by phosphorylating glycoproteins, activating the transcription of antioxidant response elements, and switching cellular energy metabolism between aerobic and anaerobic modes (129, 134–138). L-tyrosine or L-DOPA conversion to DOPAquinone is catalyzed by Tyr (51, 53, 54). Interestingly, the levels of a DOPAquinone-derived metabolite called 5-S-Cysteinyldopa (5-S-CD) are usually augmented in the urine and serum of patients with early CM stages and its levels seem to increase gradually with disease progression, peaking in metastatic CM patients. Thus, the concentration of the pheomelanogenic substrate 5-S-CD in blood and urine was suggested to be a good prognosis indicator of CM progression and treatment efficacy (139–141). Similar to 5-S-CD, the presence of Tyr in the blood of CM patients was also suggested to be a reliable prognostic biomarker to assess disease progression and treatment response (142, 143). However, the production of melanin intermediates by Tyr involves the release of cytotoxic free-radicals that induce CM cell death, which can be suppressed by both TYRP1 and TYRP2 (144). Additionally, *TYRP1* mRNA sequesters the tumor-suppressor microRNA(miR)-16 and induces CM growth. Therefore, the balance between the physiologic and harmful role of TYRP1 can depend on the abundance of its mRNA, SNPs and other miRs (145, 146). Finally, the melanosomal marker HMB45, which labels scaffold PMEL fibrils in melanosomes, has also been associated with CM diagnosis. Usually, CM patient-derived samples have diffuse or absent HMB45 expression, when compared with benign nevi (147–149). Indeed, dysplastic nevi and malignant CM show aberrant giant melanosome complexes packaged into autophagosomes, which likely undergo at least partial autophagic degradation (150–152). Moreover, transferred melanin to CM-associated keratinocytes shows heterogeneous granularity and the melanin clumps turn keratinocytes darker. These findings suggest that CM cells can transfer the melanosome/autophagosome complexes to surrounding keratinocytes, inducing the hypermelanosis usually observed in CM tumors (151, 152). Interestingly, melanosomes are metabolically-active organelles that are known to switch energy metabolism from oxidative to anaerobic glycolysis, regulate intracellular calcium signaling, and bind cations or biomolecules such as catecholamines, serotonin, and prostaglandins in melanin-recipient cells (52, 105, 136, 137, 153–155). Proteomic analyses of melanosomes illustrate their complexity and support the notion that these organelles can regulate not only melanocyte function, but also the function of other neighboring cells, including keratinocytes (52, 156). Thus, melanosomes are potential mediators of skin homeostasis and participate in diverse environmental responses. Furthermore, the presence of the supramentioned melanogenic derivatives in serum circulation of CM patients, can also act on distant sites and so affect not only skin homeostasis, but the homeostasis of other tissues and organs (52, 155).

Membrane traffic regulators involved in melanogenesis, such as Rab GTPases, were also described to have a role in CM progression (**Figure 1** and **Supplementary Table 1**). Rab22 is known to assemble in a complex with the microtubule transporter Kinesin family member 13a (KIF13a) to pull multivesicular endosome membranes, which form tubules that enable the transport of TYRP1 to melanosomes during melanogenesis (157). Interestingly, RAB22 was found to be upregulated in CM cells and patient-derived tumor samples. This Rab protein has higher expression levels in metastatic CM tumors, compared to primary tumors and was shown to induce CM proliferation, migration and invasion (158, 159). In addition, the Rab22 effector KIF13a induces membrane blebbing and increases CM cell migration (160). Rab38 and Rab4a are also regulators of both melanogenesis and CM progression. The former was described as being crucial in the transport and docking of Golgi-derived vesicles carrying the melanogenic enzymes Tyr and TYRP1 to melanosomes (161). Rab38 was also shown to increase CM invasion through MMP secretion and is found upregulated in metastatic CM cells and CM patient-derived tumor samples (158, 162). Finally, Rab4a regulates the sorting of vesicles containing Tyr and TYRP1 from endosomes to melanosomes, through microtubule-mediated transport (163). In CM, this Rab protein induces lysosome secretion, which in turn modifies tumor microenvironment and increases CM tumorigenesis and metastasis (164). Altogether, these findings demonstrate the importance of cell-to-cell communication and intracellular trafficking in regulating melanin synthesis and melanogenic machinery in CM progression.

## Melanin Secretion in Cutaneous Melanoma Invasion and Metastasis

An exacerbated expression and activity of melanogenic machinery and high levels of intracellular melanin in CM cells can contribute to the progression of this disease. However, the presence of melanin inside CM cells decreases their invasiveness and metastasis in mice, by increasing cell stiffness and reducing elasticity (34, 35, 165). This suggests that melanin negatively regulates advanced CM stages and therefore, CM cells may reduce melanogenesis and secrete higher amounts of melanin to enhance migration, invasion, intravasation into blood and lymphatic vessels, extravasation and metastasis to distal organs.

Before undergoing malignant transformation, melanocytes transport mature melanosomes to the cell periphery, followed by secretion and transfer to neighboring keratinocytes (29–31). The molecular mechanisms regulating these processes are now well characterized. Rab1a-SKIP-Kinesin 1 complex regulates anterograde melanosome transport on microtubules (166). At the periphery of melanocytes and in melanocyte dendrites, melanosomes bind to the cortical actin cytoskeleton and are kept there by the tripartite complex Rab27a-Melanophilin-Myosin Va (36, 167). Finally, melanosomes need to be tethered to the plasma membrane and primed for secretion. We reported that Rab11b interacts with the exocyst tethering complex to regulate the late steps of melanosome exocytosis (37, 168). Recently, we also found that Rab3a regulates melanin exocytosis stimulated by soluble factors released by differentiated keratinocytes, but not by undifferentiated keratinocytes (169). Furthermore, Toll-like receptor 2 (TLR2) activation was found to increase melanin synthesis and stimulate melanin secretion in a Rab11a-dependent manner (170). Another Rab GTPase - Rab17 - was shown to induce melanin release through filopodia formation (171). Myosin X and N-methyl-D-aspartate (NMDA) receptor were also described to promote melanin release through filopodia assembly (172, 173).

After secretion, melanin can be found in the dermis, where it contributes to microenvironment modulation and CM progression (73). Interestingly, several regulators of melanosome transport and secretion in melanocytes were also found to participate in CM progression (**Figure 1** and **Supplementary Table 1**). For instance, Rab1b is recruited to the Golgi apparatus in CM cells and enhances secretion of pro-invasive and pro-angiogenic proteins *in vitro* and *in vivo* (174). The tripartite complex Rab27a-Melanophilin-MyosinVa was shown to regulate CM cell migration and invasion, and higher levels of these proteins are associated with advanced CM stages in patient-derived tumor samples (38, 175). Rab27a also induces extracellular matrix (ECM) degradation by promoting the secretion of vesicles carrying the membrane type 1-MMP (39). Interestingly, these vesicles contain TYRP1 and the multivesicular body (MVB) marker CD63, suggesting a functional crosstalk between MVBs and melanosome traffic machinery in CM progression (39). Another secretory Rab - Rab3d - enhances proliferation, migration and invasiveness of several CM cell lines *in vitro* (176, 177). Rab3d has more than 80% sequence homology with Rab3a, which we described as a regulator of melanosome exocytosis (169, 178, 179). Moreover, we described that Rab3a regulates lysosome exocytosis, which is involved in plasma membrane repair, among other functions (180). Indeed, in CM cells, lysosome exocytosis enhances plasma membrane repair after UVr exposure, as well as the release of the lysosomal proteases Cathepsin B and K, which in turn degrade the ECM, thereby promoting CM invasion and metastasis *in vitro* and *in vivo* (181–184). Rab11 was also shown by us to cooperate with Rab3a in plasma membrane repair in HeLa cells (185). Curiously, both Rab11a and Rab11b regulate melanin release from melanocytes and stimulate EMT in CM cells by switching the expression of E-cadherin to N-cadherin at the cell surface (168, 170, 186, 187). Furthermore, the exocyst subunits Exo70 and Sec8, which were described by us to cooperate with Rab11b to secrete melanin in melanocytes, also increase CM invasiveness through invadopodia formation and release of MMPs (37, 40). These proteins, found to induce melanin secretion by assembling filopodia in melanocytes, were also shown to have a role in CM progression. Rab17 promotes CM growth *in vivo* (146), whereas Myosin X has a crucial role in CM proliferation and metastasis *in vivo*, and its higher expression is associated with advanced CM stages in patient-derived tumor samples (188). Finally, NMDA receptor promotes proliferation, migration and invasion of CM cells, and enhanced NMDA receptor activation is associated with faster disease progression and lower overall survival (189, 190).

Hence, the molecular machinery used by melanocytes for melanosome transport and secretion seems to have a dual role by simultaneously regulating the exocytosis of melanin and other factors such as MMPs, to promote CM progression. However, more studies are required to assess the impact of melanin secretion in CM, and better understand the molecular mechanisms involved, to design efficient therapies to block CM aggressiveness.

## The Role of Melanin in Immune System Modulation

Tumor microenvironment is known to influence cancer aggressiveness and therapy resistance. CM is considered one of the most immunogenic types of neoplasms, with a high immunomodulatory potential that enables it to suppress and escape immune recognition (23). Besides their important role in skin pigmentation, there is evidence that melanocytes are immunocompetent cells (191–193). Indeed, they show resemblance with antigen−presenting cells (APCs) in their dendritic morphology and expression of APC surface markers such as Cluster of differentiation (CD) 40, Human leukocyte antigen DR (HLA-DR), Intercellular adhesion molecule 1 (ICAM-1), and TLRs (191, 192). IFN-γ treatment leads to the overexpression of CD40 and ICAM-1 by cultured melanocytes, promoting T lymphocyte proliferation and IL-12 secretion. This is thought to be one of the skin’s defense mechanisms to prevent harm from external pathogens, which is also supported by the phagocytic ability of melanocytes (192, 193). In this context, it is reasonable to assume that melanin could also play an important role in this immunomodulation, influencing CM progression and therapy response.

Indeed, the presence of intracellular melanin increases chemotherapy and radiotherapy resistance of CM cells, suggesting melanogenesis as a possible therapeutic target for the management of advanced CM stages (194, 195). Moreover, the outcome of radiotherapy and chemotherapy is impaired in patients with pigmented CM metastases, resulting in the reduction of their overall survival, when compared with patients with amelanotic metastases (196, 197). Interestingly, monoclonal antibodies targeting melanin were shown as promising radioimmunotherapy agents against metastatic CM (198). On the other hand, melanocyte-related proteins involved in melanogenesis, such as Tyr, TYRP1, TYRP2, and Melan-A have been shown to be targets of anti-melanoma cytotoxic T lymphocytes that, when successful, lead to the destruction of epidermal melanocytes (199–202). In fact, this process has been proposed as one of the causes of vitiligo - an auto-immune disorder characterized by the loss of epidermal melanocytes, leading to depigmented skin patches - in some CM patients, an indication of a strong anti-melanoma immune reaction (203–205). Indeed, there is an accumulation of CD8^+^ T lymphocytes at the margins of these depigmented areas. Vitiligo-like depigmentation can also be an adverse effect of immune−checkpoint inhibitor therapy, but this depigmentation is associated with better clinical outcomes (206). However, due to their high plasticity and immunomodulatory potential, CM cells are able to develop mechanisms of immune evasion and suppression, some of them linked to melanogenesis.

Some melanogenesis precursors, such as α-MSH, are linked to immune tolerance and anti-inflammation (76, 207, 208). This hormone is secreted upon exposure to UVr, binding to MC1R at the surface of melanocytes and triggering a cascade of events that enhances melanogenesis (83, 84, 209) (**Supplementary Table 1**). However, human monocytes, macrophages, dendritic cells, endothelial cells, fibroblasts and T lymphocytes can also express melanocortin receptors, such as MC1R and MC5R (76, 210–212). Binding of α-MSH to MC1R on the surface of monocytes or macrophages can inhibit Nuclear factor kappa B (NF-κB) translocation to the nucleus in a cAMP−dependent process, and consequently inhibit production of pro-inflammatory cytokines, such as IL-1, IL-2, IL−6, IL−13, as well as TNF-α and IFN-γ (207, 208). IL-6 seems to inhibit the early stages of CM formation, but has the opposite effect in advanced metastatic CM, being indicative of poor prognosis (213–216). α-MSH is also thought to inhibit NF-κB in T lymphocytes, leading to a decrease in IFN-γ production, whilst triggering upregulation of the anti-inflammatory cytokine IL-10 and Transforming growth factor (TGF)-β, possibly through cAMP responsive element binding protein (CREB). Thus, modulation of cAMP signaling by α-MSH can convert effector T lymphocytes into regulatory T lymphocytes (212, 217, 218). Furthermore, α-MSH was shown to also upregulate the production of IL-10 in macrophages and monocytes, which might assist the expansion of CM-initiating cells (216, 219). Inhibition of nitric oxide (NO) production by macrophages in a NF-κB dependent manner further supports the immunosuppressive role of α−MSH (76, 208, 220) (**Figure 1**).

Another player in the melanogenesis cascade that has been implicated in immunosuppression is the melanogenic substrate L-DOPA. *In vitro* data shows that L-DOPA inhibits lymphocyte proliferation and activity by preventing DNA synthesis and cytokine expression (195, 221). Thus, pigmented human CM cells seem to more easily evade elimination by T lymphocytes, a feature that is lost upon melanogenesis inhibition (195). These observations further support the hypothesis that melanin intermediates assist in a more immunosuppressive microenvironment, which might contribute to CM cell proliferation and therapy resistance (**Figure 1**). However, the molecular mechanisms underlying this immunosuppressive role of L-DOPA are still poorly explored. Evidence suggests that T lymphocytes are able to release dopamine, of which L-DOPA is an important precursor. Dopamine is thought to be a powerful immunomodulator, with different immune subsets expressing dopamine receptors that trigger various suppression and activation pathways (222).

The induction of melanogenesis in CM cell lines also seems to lead to higher Hypoxia-inducible factor 1 subunit alpha (HIF-1α) levels (223). Indeed, higher pigmentation levels in CM are correlated with the upregulation of HIF-1α and HIF-1α-dependent genes, such as *VEGFA*, *Solute carrier family 2 member 1* (*SLC2A1*), *SLC16A7*, *SLC9A1*, *Pyruvate dehydrogenase kinase 1* (*PDK1*), *Fructose-bisphosphate aldolase* (*ALDOA*), *Lactate dehydrogenase A* (*LDHA*) and *Hexokinase 2* (*HK2*), involved in glycolysis, angiogenesis and stress response, ultimately contributing to increased CM aggressiveness (223). Melanogenesis also seems to upregulate *TGFB2*, *IL5*, *IL10* and *IL17* expression by CM cells, but its impact on cytokine expression was found to be overall limited (223). On the other hand, an *in vivo* study showed that TGFβ2 induces hypopigmentation in CM cells, and inhibits dendrite formation, leading to higher cell motility. TGFβ2 is also upregulated in non-pigmented CM cells, being considered an MITF antagonist (224). Recently, Pawlikowska *et al.* have also published evidence that melanogenesis might promote an immunosuppressive CM microenvironment, corroborating previous studies. Using a co-culture system of pigmented CM cells with peripheral blood mononuclear cells (PBMCs), the authors showed that melanogenesis inhibition through Tyr inhibitors induces the expression of proinflammatory cytokines by PBMCs, such as IL-1β, IL-2, IL-6 and IL-12 (225). IL-12 has been associated with anti-CM activity, and its suppression is considered pro-tumoral in most cases (226, 227).

Interestingly, one study has correlated synthetic melanin levels with the inhibition of proinflammatory cytokine production by human monocytes, possibly indicating that melanin secretion itself, and not just melanogenesis intermediates, might have an important immunomodulatory role. Synthetic melanin suppresses TNF, IL-1β and IL-6 production by interfering with post-transcriptional events (74). Finally, after secretion from CM cells, melanin was found to transform dermal fibroblasts in cancer-associated fibroblasts (CAFs), suppressing the immune system and promoting tumor angiogenesis, thus sustaining CM progression and metastasis (73).

Even though evidence supporting the immunosuppressive role of melanogenesis has emerged in the last decades, there is still much to explore when it comes to its contribution to the tumor microenvironment and, consequently, to CM progression. Thus far, research suggests that melanin synthesis and secretion contribute to the inhibition of proinflammatory cytokine expression by monocytes, macrophages and T lymphocytes, with consequent suppression of T lymphocyte proliferation and activity, whilst also stimulating the activity of regulatory T lymphocytes in the tumor vicinity (**Figure 1**). This would promote an immunosuppressive microenvironment, possibly supporting CM aggressiveness and tumor evasion. However, further research must be conducted to support these hypotheses, as they are sustained mainly by few *in vitro* studies. More specifically, there is still a gap in the knowledge concerning the effect of melanogenesis and melanin secretion on the recruitment of different subsets of immune cell populations to the tumor microenvironment, a phenomenon that is known to impact CM progression and immunotherapy response. To our knowledge, there are currently no studies that directly relate melanin production and secretion with immunotherapy response through the immunomodulation of the CM microenvironment.

## Conclusions and Future Perspectives

Recent evidence has implicated melanin in the promotion of CM progression and capacity of CM cells to adapt throughout distinct CM stages, *i.e.*, CM plasticity. Indeed, several known molecules involved in melanogenesis and melanin secretion play distinct and pivotal roles in CM tumorigenesis and progression (**Supplementary Table 1**). Therefore, the therapeutic targeting of melanogenesis and melanin secretion molecular machinery may be an efficient approach to impair CM cell migration, invasion and, importantly, metastasis. However, considering the complexity and variety of the roles that melanin plays in CM progression, much remains to be understood regarding its impact on CM pathology, therefore warranting more studies. The literature suggests a role for melanin in the malignant transformation of melanocytes and CM cell immortalization, but its influence in CM progression remains poorly understood and controversial, as melanin seems to have distinct roles depending on the CM stage. Several studies implicated melanogenesis and melanin release in CM plasticity in the course of the disease. Indeed, changes in the molecular machinery responsible for regulating melanin synthesis in melanocytes have been linked to the phenotypic switch from proliferative to invasive CM states (**Supplementary Table 1**). However, the presence of melanin inside CM cells seems to decrease their invasiveness and metastasis, suggesting that enhanced melanin secretion is crucial for CM cell invasion and microenvironment modulation. Furthermore, several known regulators of melanin transport in and secretion from melanocytes also play a role in CM progression and modulation of tumor microenvironment (**Supplementary Table 1**). Therefore, a double therapeutic approach enhancing melanin synthesis and blocking melanin secretion could impair CM transition to advanced stages, which leads to a reduced patient overall survival. Thus, further elucidation of this dual role for melanin synthesis and release in CM progression might provide novel therapeutic strategies to ensure the specific targeting of CM cells, depending on the disease stage, as an attempt to improve CM patient overall survival and quality of life.

Additionally, one must consider the potential role of melanin synthesis and especially melanin secretion in the modulation of CM microenvironment. Indeed, it is known that the interaction between CM cells and the immune system has a strong influence on CM prognosis and response to immunotherapy. As discussed previously, evidence suggests that some melanogenesis regulators and perhaps even melanin secretion itself, might promote an immunosuppressive microenvironment, which would further support CM aggressiveness and consequent poor prognosis. However, research exploring the immunomodulatory potential of melanin has been stagnant in recent years and, consequently, the molecular mechanisms underlying these processes are still vastly underexplored. Research in this field has relied on an abundance of different CM cell lines as models (**Supplementary Table 2**), and the range in pigmentation levels and different genetic backgrounds has brought relevant insights into the dual role of melanin in the pathogenesis of CM. However, the use of these models does not seem to be enough to properly explore the complex interactions between melanin and its regulators and immune cells in the tumor vicinity. Recently, 3D cell culture models have evolved to become more reliable and intricate, and could in the future recapitulate the most important CM features, including the tumor microenvironment. These types of methodological advancements will be extremely advantageous and are crucial to better understand the impact of melanin secretion on the modulation of the immune microenvironment and how it correlates to immunotherapy response.

## Author Contributions

All authors listed have made a substantial, direct and intellectual contribution to the work, and approved it for publication.

## Funding

LC is funded by an FCT PhD fellowship (2020.08812.BD). AT is funded by an FCT PhD fellowship (2021.06204.BD). MP is funded by Liga Portuguesa Contra o Cancro – Núcleo Regional do Sul (LPCC-NRS). DB lab is supported by Fundação para a Ciência e a Tecnologia (FCT) through grant PTDC/BIA-CEL/29765/2017. This publication was funded by the iNOVA4Health - UIDB/04462/2020 and UIDP/04462/2020, a program financially supported by FCT/Ministério da Educação e Ciência through national funds and co-funded by FEDER under the PT2020 Partnership Agreement. The funders were not involved in the study design, collection, analysis, interpretation of data, the writing of this article or the decision to submit it for publication.

## Conflict of Interest

The authors declare that the research was conducted in the absence of any commercial or financial relationships that could be construed as a potential conflict of interest.

## Publisher’s Note

All claims expressed in this article are solely those of the authors and do not necessarily represent those of their affiliated organizations, or those of the publisher, the editors and the reviewers. Any product that may be evaluated in this article, or claim that may be made by its manufacturer, is not guaranteed or endorsed by the publisher.

## Abbreviations

CM, Cutaneous melanoma; MEK, Mitogen-activated protein kinase; UVr, Ultraviolet radiation; ROS, Reactive oxygen species; RNS, Reactive nitrogen species; CPDs, Cyclobutane pyrimidine dimers; Tyr, Tyrosinase; TYRP1, Tyrosinase-related protein 1; TYRP2, Tyrosinase-related protein 2; α-MSH, α-Melanocyte stimulating hormone; MC1R, Melanocortin-1 receptor; cAMP, Cyclic adenosine monophosphate; MITF, Microphthalmia-associated transcription factor; SNP, Single-nucleotide polymorphism; SCF, Stem-cell factor; Rab, Ras-like proteins in brain; EMT, Epithelial-mesenchymal transition; MMPs, Matrix metalloproteinases; miR, MicroRNA; 5-S-CD, 5-S-cysteinyldopa; KIF13a, Kinesin family member 13a; TLR2, Toll-like receptor 2; NMDA, N-methyl-D-aspartate; ECM, Extracellular matrix; MVB, Multivesicular body; MAPK, Mitogen-activated protein kinase; ERK, Extracellular signal-regulated kinase; AKT, Protein kinase B; PI3K, Phosphoinositide 3-kinases; IL, Interleukin; IFN, Interferon; TNF, Tumor necrosis factor; NF-κB, Nuclear factor kappa B; HIF-1α, Hypoxia inducible factor 1 subunit alpha; TGF, Transforming growth factor; *VEGFA*, Vascular endothelial growth factor A; *SLC2A1*, Solute carrier family 2 member 1; *SLC16A7*, Solute carrier family 16 member 7; *SLC9A1*, Solute carrier family 9 member A1; *PDK1*, Pyruvate dehydrogenase kinase 1; *ALDOA*, Fructose-bisphosphate aldolase; *LDHA*, Lactate dehydrogenase A; *HK2*, Hexokinase.
