# Supplementary material for: The Dark Side of Melanin Secretion in Cutaneous Melanoma Aggressiveness
Source: Front Oncol. 2022 May 10;12:887366. doi: 10.3389/fonc.2022.887366 (PMC9128548; doi:10.3389/fonc.2022.887366)
Supplement: Supplementary file 1 [file Table_1.docx]

**Supplementary Table 1. Melanoma-related genes involved in melanin synthesis, transport and secretion.**

| **Gene name** | **Protein name** | **Accession number** | **Functions in pigmentation** | **Functions in CM progression** | **References** |
| --- | --- | --- | --- | --- | --- |
| *MC1R* | Melanocortin 1 Receptor (MC1R) | [NM_002386](https://www.ncbi.nlm.nih.gov/nuccore?term=NM_002386) | MC1R is activated upon recognition of the keratinocyte-derived α-MSH soluble factor and stimulates melanin synthesis via MITF-target genes. MC1R polymorphisms are directly associated with the ratio of eumelanin/pheomelanin produced. | MC1R enhances CM proliferation and survival through the MAPK/ERK-signaling pathway, as well as migratory capacity by controlling the expression of the transmembrane protein Sidecan-2. MC1R may have a role in the CM microenvironment, since monocytes, macrophages, dendritic cells, endothelial cells and fibroblasts also express this receptor. In monocytes and macrophages, MC1R activation inhibits the production of several pro-inflammatory cytokines. | 83-90; 76; 207-210 |
| *KIT* | c-Kit receptor tyrosine kinase (c-Kit) | [NM_000222](https://www.ncbi.nlm.nih.gov/nuccore?term=NM_000222) | c-kit is activated upon recognition of the keratinocyte- and fibroblast-derived SCF and stimulates melanin synthesis via MITF-target genes. It can also be transactivated by MC1R. | c-Kit receptor mutants stimulate CM proliferation and survival through MAPK/ERK and PI3K/AKT signaling pathways. | 89; 91-93 |
| *EDNRB* | Endothelin receptor type B (ETBR) | [NM_000115](https://www.ncbi.nlm.nih.gov/nuccore?term=NM_000115) | ETBR is activated upon recognition of the keratinocyte-derived endothelin soluble factor and stimulates melanin synthesis via MITF-target genes. | ETBR mutants stimulate CM proliferation, survival, neoangiogenesis, migration, EMT and invasion. | 94-98 |
| *MET* | c-Met receptor tyrosine kinase (c-Met) | [NM_000245](https://www.ncbi.nlm.nih.gov/nuccore?term=NM_000245) | c-Met stimulates melanin synthesis via MITF-target genes upon recognition of the keratinocyte- and fibroblast-derived hepatocyte growth factor. | c-Met mutants increase CM proliferation and survival through the MAPK/ERK pathway, as well as migration and invasiveness through ROS-mediated signaling. | 99; 100 |
| *MITF* | Microphthalmia-associated transcription factor (MITF) | [NM_198159](https://www.ncbi.nlm.nih.gov/nuccore?term=NM_198159) | Master transcription factor regulating the expression of melanogenic enzymes like Tyr, as well as melanocyte dendricity, proliferation and differentiation. | Hyperactived MITF increases melanin synthesis and proliferation rates in CM cells, while a reduced MITF activation is involved in the phenotypic switch from proliferation to invasion in advanced CM stages. MITF is also associated with intratumor heterogeneity, CM plasticity and increased therapeutic resistance.  TGFβ2 is considered an MITF antagonist and induces hypopigmentation in CM cells, inhibits dendrite formation and increases CM cell motility. | 29; 30; 83; 84; 119-127; 224 |
| *TYR* | Tyrosinase (Tyr) | [NM_000372](https://www.ncbi.nlm.nih.gov/nuccore?term=NM_000372) | Tyr catalyzes L-tyrosine conversion to L-DOPA, the first step in melanin synthesis. | The presence of Tyr in the blood of CM patients was suggested to be a reliable prognostic parameter to assess disease progression and treatment response. Moreover, CM cells with high Tyr activity suppress the release of pro-inflammatory cytokines and more easily evade elimination by T lymphocytes. | 51; 53; 54; 143; 144; 218; 222 |
| *TYRP1* | Tyrosinase-related protein 1 (TYRP1) | [NM_000550](https://www.ncbi.nlm.nih.gov/nuccore?term=NM_000550) | TYRP1 controls eumelanogenesis rate, by fine tuning Tyr activity, even though it is not required for pheomelanogenesis. | Tyr involves the release of cytotoxic free-radicals that induce CM cell death, which can be suppressed by TYRP1. *Its* mRNA also sequesters the tumor-supressor miR-16 and induces CM growth. | 51; 53; 55; 62; 144-146 |
| *TYRP2* | Tyrosinase-related protein 2 (TYRP2) | [NM_001129889](https://www.ncbi.nlm.nih.gov/nuccore?term=NM_001129889) | TYRP2 controls eumelanogenesis rate by fine-tuning Tyr activity, even though it is not required for pheomelanogenesis. | Tyr involves the release of cytotoxic free-radicals that induce CM cell death, which can be suppressed by TYRP2. | 51; 53; 55; 62; 144 |
| *PMEL* | Premelanosome protein (PMEL) | [NM_006928](https://www.ncbi.nlm.nih.gov/nuccore?term=NM_006928) | PMEL forms scaffold fibrils inside melanosomes, onto which melanin deposits. | PMEL has diffuse or absent expression in CM patient-derived samples, when compared with benign nevi samples. | 147-149 |
| *RAB22* | Ras-like in brain 22 (Rab22) | [NM_020673](https://www.ncbi.nlm.nih.gov/nuccore?term=NM_020673) | Rab22 assembles in a complex with the microtubule-dependent molecular motor KIF13a, to pull multivesicular endosome membranes that form tubules, enabling the transport of TYRP1 to melanosomes during melanogenesis. | RAB22 is upregulated in CM cells and patient-derived tumor samples. It has higher expression levels in metastatic CM tumors, compared to primary tumors and was shown to induce CM proliferation, migration and invasion. | 157-159 |
| *KIF13A* | Kinesin family member 13a (KIF13a) | [NM_001105566](https://www.ncbi.nlm.nih.gov/nuccore?term=NM_001105566) | KIF13a is a Rab22 effector recruited to form tubules that allow the transport of TYRP1 to melanosomes. | KIF13a induces membrane blebbing and increases CM cell migration. | 157; 160 |
| *RAB38* | Ras-like in brain 38 (Rab38) | [NM_022337](https://www.ncbi.nlm.nih.gov/nuccore?term=NM_022337) | Rab38 transports and docks Golgi-derived vesicles carrying the melanogenic enzymes Tyr and TYRP1 to melanosomes during melanin synthesis. | Rab38 is upregulated in metastatic CM cells and CM patient-derived tumor samples. It increases CM invasion through MMP secretion. | 158; 161; 162 |
| *RAB4A* | Ras-like in brain 4a (Rab4a) | [NM_004578](https://www.ncbi.nlm.nih.gov/nuccore?term=NM_004578) | Rab4a coordinates the sorting of vesicles containing Tyr and TYRP1 from endosomes to melanosomes during melanin synthesis. | Rab4a induces lysosome secretion in CM, which in turn modifies tumor microenvironment and increases CM tumorigenesis and metastasis. | 163; 164 |
| *RAB1A/B* | Ras-like in brain 1a/b (Rab1a/b) | [NM_004161](https://www.ncbi.nlm.nih.gov/nuccore?term=NM_004161)  [NM_030981](https://www.ncbi.nlm.nih.gov/nuccore?term=NM_030981) | Rab1a regulates anterograde melanosome transport on microtubules. | Rab1b enhances secretion of pro-invasive and pro-angiogenic proteins from CM cells *in vitro* and *in vivo*. | 166; 174 |
| *RAB27A* | Ras-like in brain 27a (Rab27a) | [NM_004580](https://www.ncbi.nlm.nih.gov/nuccore?term=NM_004580) | Rab27a tethers melanosomes to the cortical actin cytoskeleton at the periphery and dendrites of melanocytes. | Rab27a regulates CM cell migration and invasion through MMP release and extracellular matrix degradation. Higher levels of this protein are associated with advanced CM stages in patient-derived tumor samples. | 36; 38; 39; 167; 175 |
| *MYO5* | Myosin V | [NM_000259](https://www.ncbi.nlm.nih.gov/nuccore?term=NM_000259) | Myosin V forms a complex with Rab27a and transports melanosomes on the cortical actin cytoskeleton of melanocytes. | Myosin V regulates CM cell migration and invasion, and higher levels of this protein are associated with advanced CM stages in patient-derived tumor samples. | 36; 38; 167; 175 |
| *MLPH* | Melanophilin (SLAC2-A) | [NM_024101](https://www.ncbi.nlm.nih.gov/nuccore?term=NM_024101) | Melanophilin is an adaptor protein that links Rab27a and Myosin V, allowing the transport of melanosomes on the cortical actin cytoskeleton. | Melanophilin regulates CM cell migration and invasion, and higher levels of this protein are associated with advanced CM stages in patient-derived tumor samples. | 36; 38; 167; 175 |
| *RAB11A/B* | Ras-like in brain 11a/b (Rab11a/b) | [NM_001206836](https://www.ncbi.nlm.nih.gov/nuccore?term=NM_001206836)  [NM_004218](https://www.ncbi.nlm.nih.gov/nuccore?term=NM_004218) | Rab11b regulates the late steps of melanosome exocytosis by interacting with the exocyst tethering complex. Rab11a is involved in the melanin secretion mediated by TLR2. | Rab11a and Rab11b stimulate EMT in CM cells by switching the expression of E-cadherin to N-cadherin at the cell surface. | 37; 168; 170; 186; 187 |
| *EXOC7* | Exocyst complex component 7 (Exo70) | [NM_015219](https://www.ncbi.nlm.nih.gov/nuccore?term=NM_015219) | Exo70 tethers melanosomes to the plasma membrane,as part of the exocyst tethering complex. | Exo70 increases CM invasiveness through invadopodia formation and release of MMPs. | 37; 40 |
| *EXOC4* | Exocyst complex component 4 (Sec8) | [NM_021807](https://www.ncbi.nlm.nih.gov/nuccore?term=NM_021807) | Sec8 tethers melanosomes to the plasma membrane, as part of the exocyst tethering complex. | Sec8 increases CM invasiveness through invadopodia formation and release of MMPs. | 37; 40 |
| *RAB3A/D* | Ras-like in brain 3a/d (Rab3a/d) | [NM_002866](https://www.ncbi.nlm.nih.gov/nuccore?term=NM_002866)  [NM_004283](https://www.ncbi.nlm.nih.gov/nuccore?term=NM_004283) | Rab3a stimulates melanin exocytosis induced by keratinocyte-conditioned medium. | Rab3d enhances proliferation, migration and invasiveness of several CM cell lines. | 169; 176; 177 |
| *RAB17* | Ras-like in brain 17 (Rab17) | [NM_022449](https://www.ncbi.nlm.nih.gov/nuccore?term=NM_022449) | Rab17 induces melanin release through filopodia formation. | Rab17 promotes CM growth *in vivo*. | 146; 171 |
| *MYOX* | Myosin X | [NM_012334](https://www.ncbi.nlm.nih.gov/nuccore?term=NM_012334) | Myosin X promotes melanin release through filopodia assembly. | Myosin X has a crucial role in CM proliferation and metastasis *in vivo*, and its higher expression is associated with advanced CM stages in patient-derived tumor samples. | 173; 188 |
| *GRIN1/GRIN2A* | Glutamate ionotropic receptor N-methyl-D-aspartate type subunit 1/2a (NMDA receptor) | [NM_007327](https://www.ncbi.nlm.nih.gov/nuccore?term=NM_007327)  [NM_000833](https://www.ncbi.nlm.nih.gov/nuccore?term=NM_000833) | NMDA receptor induces melanin release through filopodia formation. | NMDA receptor promotes proliferation, migration and invasion of CM cells, and enhanced NMDA receptor activation is associated with faster disease progression and shorter overall survival. | 172; 189; 190 |
