# Supplementary material for: The Dark Side of Melanin Secretion in Cutaneous Melanoma Aggressiveness
Source: Front Oncol. 2022 May 10;12:887366. doi: 10.3389/fonc.2022.887366 (PMC9128548; doi:10.3389/fonc.2022.887366)
Supplement: Supplementary file 2 [file Table_2.docx]

**Supplementary Table 2. Cutaneous melanoma cell lines and their characteristics.**

| **Cell line** | **RRID number** | **Species** | **CM stage and isolation site** | **Pigmentation status** | **Other cellular and molecular features** |
| --- | --- | --- | --- | --- | --- |
| MNT-1 | CVCL_5624 | Human | N.D. stage/ Lymph node metastasis | Highly pigmented | N.D. |
| MUG-Mel2 | CVCL_JQ50 | Human | Stage IV/Cutaneous metastasis | Highly pigmented | NRAS Q61R |
| SK-MEL-1 | CVCL_0068 | Human | Stage IV/Thoracic duct metastasis | Pigmented | BRAF V600E  CTNN S33C  Spherical, non-adherent |
| SK-MEL-10 | CVCL_6020 | Human | N.D. Stage/Primary tumor | Non-pigmented | FGFR2 A625Q  NRAS Q61A |
| SK-MEL-25 | CVCL_6028 | Human | N.D. Stage/Metastasis | Pigmented | N.D. |
| SK-MEL-28 | CVCL_0526 | Human | N.D. Stage/Axillary lymph node metastasis | Non-pigmented | BRAF V600E  CDK4 R24C  EGFR P753S  PTEN T167A  TP53 L145R |
| A375 | CVCL_0132 | Human | N.D. stage/Primary tumor | Non-pigmented | BRAF V600E  CDKN2A G75V  CDKN2A G83V |
| G-361 | CVCL_1220 | Human | N.D. Stage/Primary tumor | Pigmented | BRAF V600E |
| SW-489 | CVCL_1E89 | Human | N.D. Stage/Primary tumor | Non-pigmented | N.D. |
| Malme-3M | CVCL_1438 | Human | N.D. Stage/Lung metastasis | Pigmented | BRAF V600E  CDKN2A deletion |
| Mel-2a | CVCL_A759 | Human | N.D. Stage/Axillary lymph node metastasis | Non-pigmented | N.D. |
| Mel-57 | CVCL_4454 | Human | N.D. Stage/Cutaneous metastasis | Non-pigmented | N.D. |
| Mel-67 | CVCL_WG85 | Human | N.D. Stage/Metastasis | Pigmented | N.D. |
| WM35 | CVCL_0580 | Human | Stage I/Primary tumor | Non-pigmented | BRAF V600E |
| WM1789 | CVCL_6792 | Human | Stage I/Primary tumor | Pigmented | BRAF K601E  PTEN deletion |
| WM793B | CVCL_8787 | Human | Stage II/Primary tumor | Pigmented | BRAF V601E  PTEN W274Ter  CDK4 K22Q |
| WM3268V | CVCL_L031 | Human | Stage III/ Primary tumor | Pigmented | NRAS Q61K |
| WM853-2 | CVCL_C282 | Human | Stage III/Primary tumor | Non-pigmented | BRAF V600E  TP53 deletion |
| WM75 | CVCL_7156 | Human | Stage III/Primary tumor | Pigmented | BRAF V600E  CDKN2AE88K |
| WM115 | CVCL_0040 | Human | Stage IV/Primary tumor | Non-pigmented | BRAF V600D  PTEN deletion |
| WM278 | CVCL_6473 | Human | N.D. Stage/Primary tumor | Non-pigmented | BRAF V600E  PTEN deletion |
| WM3211 | CVCL_6797 | Human | N.D. Stage/Acral lentiginous melanoma; lymph node metastasis | Non-pigmented | CDKN2A Q50Ter  KIT L576P  TP53 C242G |
| WM164 | CVCL_7928 | Human | Stage IV/ right upper arm metastasis | Non-pigmented | BRAF V600E |
| WM1158 | CVCL_6785 | Human | Stage III/Lymph node metastasis | Pigmented | BRAF V600E |
| WM373 | CVCL_C277 | Human | Stage III/Cutaneous metastasis derived from WM75 primary tumor | Pigmented | BRAF V600E |
| WM165 | CVCL_RN55  CVCL_L033 | Human | Stage IV/Lymph node metastasis derived from WM115 primary tumor | Non-pigmented | BRAF V600D  PTEN deletion |
| WM239A | CVCL_6795 | Human | Stage IV/Lymph node metastasis derived from WM115 primary tumor | Non-pigmented | BRAF V600D  PTEN deletion |
| WM266-4 | CVCL_2765 | Human | Stage IV/Cutaneous metastasis derived from WM115 primary tumor | Non-pigmented | BRAF V600D  PTEN deletion |

N.D. Not determined
